# The Effect of Brief Mindfulness Meditation on Attention Networks in University Students with Problematic Short-Video Use

**DOI:** 10.3390/bs16091487

**Published:** 2026-08-25

**Authors:** Tingting Liu, Siti Hajar Binti Halili, Norharyanti Binti Mohsin, Bingqi Li

**Affiliations:** 1 Faculty of Education, Universiti Malaya, Kuala Lumpur 50603, Malaysiayantimohsin@um.edu.my (N.B.M.); 2 Faculty of Educational Sciences and Technology, Universiti Teknologi Malaysia, Skudai, Johor Bahru 81300, Johor, Malaysia

**Keywords:** brief mindfulness meditation, problematic short-video use, Attention Network Test, executive control

## Abstract

To examine the effects of brief mindfulness meditation on attention network function in university students with problematic short-video use, participants were screened using the SPAI and an adapted IAT and assigned to an intervention or control group. The intervention group received 4 weeks of brief mindfulness meditation, whereas the control group received no training. Both groups completed the Attention Network Test before and after the intervention. MANCOVA examined group differences in alerting, orienting, and executive control while controlling for baseline performance, age, and sex. Follow-up ANCOVAs were exploratory and Holm-adjusted. The overall multivariate group effect was not significant. Exploratory analyses showed no significant between-group differences in alerting or orienting, whereas the intervention group showed a significantly lower executive control effect than the control group after Holm adjustment. Descriptive RT and ACC patterns across the five ANT conditions did not indicate a consistent improvement in task performance. Taken together, brief mindfulness meditation did not produce an overall improvement in attention network function. However, exploratory findings suggest that it may be associated with a lower conflict-processing cost in executive control. This finding should be interpreted cautiously and requires further verification.

## 1. Introduction

Short videos are a form of audiovisual content primarily distributed through mobile social media platforms, with typical platforms or features including TikTok, Douyin, Instagram Reels, and YouTube Shorts. Compared with the continuous narrative formats commonly used in television, films, and general long-form videos, short videos are typically characterized by brief content units, relatively high information density, rapid content switching, and immediate interactive feedback. Short-video platforms also commonly employ vertical full-screen presentation, continuous scrolling, and algorithm-based personalized recommendation, enabling users to quickly access and switch between content with relatively low operational effort. Technical features such as recommendation accuracy, content novelty, and perceived ease of use may further enhance user engagement and the tendency toward continued use (Bhandari & Bimo, 2022; Roberts & David, 2025).

For adolescent and young users, entertainment, information seeking, social interaction, trend following, and temporary relief from daily stress are important motivations for using short videos (Jung et al., 2025; Schellewald, 2023). However, the appeal of short videos is not exclusive to younger populations. Middle-aged and older users may also use short videos for purposes such as information seeking and entertainment, and the motivations and patterns of engagement across age groups may be influenced by factors such as digital experience and adaptation to platforms (Menon, 2022; Yu et al., 2024). Meanwhile, with the rapid proliferation of short-video platforms, problematic short-video use and its potential adverse consequences have gradually attracted research attention (Jain et al., 2025).

Existing studies commonly use terms such as “short-video addiction,” “problematic short-video use,” or “short-video addiction tendency” to describe a state in which individuals have difficulty controlling their short-video use and consequently experience impairment in learning, work, social interaction, or psychological functioning. However, there is currently no fully consistent operational definition or dedicated measurement standard for problematic video-streaming use; therefore, frequent short-video use should not be directly equated with addiction in the clinical sense (Rahat et al., 2022). Research on problematic video-streaming use, TikTok use, and general problematic Internet use has been conducted across different cultural contexts in Asia, Europe, and the Americas, suggesting that uncontrolled digital media use may share certain cross-cultural characteristics while also being influenced by cultural norms, platform environments, and measurement approaches (Lopez-Fernandez, 2015; Smith & Short, 2022). Social discourses surrounding media addiction also reflect different societal understandings of technology, self-discipline, productivity, and digital well-being (Vanden Abeele & Mohr, 2021). Therefore, problematic short-video use should be defined cautiously by considering the specific platform, actual usage behavior, and associated functional impairment. In this study, university students who exhibit relatively high levels of problematic short-video use tendency during screening are referred to as “university students with problematic short-video use,” and this term does not indicate that they meet the diagnostic criteria for clinical addiction.

Problematic short-video use may develop through the combined influence of platform characteristics, individual vulnerability factors, and the social environment. Personalized recommendations, continuous content feeds, immediate feedback, and constantly updated novel content may reduce the operational effort required to continue viewing and enhance immersion and the tendency toward prolonged viewing (Zhang et al., 2024). Lower distress tolerance, the need for negative emotion regulation, boredom proneness, and weaker self-control may increase the risk of problematic use (Hu & Huang, 2024; Yao et al., 2023); needs for social interaction, social support, and peer pressure may also influence short-video use behavior (Deng et al., 2026; Yang et al., 2022). The repeated interaction among platform cues, individual emotional and cognitive responses, and control capacity is consistent with the interaction among personal characteristics, affective and cognitive responses, and executive control processes emphasized in the I-PACE model (Brand et al., 2019).

The above processes may also influence individuals’ patterns of attention. Continuous exposure to brief, novel, and rapidly switching content may reinforce externally stimulus-driven attentional allocation, causing individuals to frequently reorient their attention and increasing the demands on sustained attention, reallocation of attentional resources, inhibition of irrelevant stimuli, and conflict monitoring. Existing studies have found that higher levels of problematic short-video use tendency are associated with lower self-control, attentional control, and indicators related to executive control (Al-Leimon et al., 2025; Xie et al., 2023; Yan et al., 2024). However, the existing evidence is primarily derived from cross-sectional and correlational studies and therefore cannot determine whether problematic short-video use leads to persistent declines in attentional functioning, nor can it establish whether related attentional difficulties can be improved through short-term intervention.

Attention is not a single ability but comprises multiple interconnected yet relatively distinct functional subsystems. Posner and Petersen (1990) proposed that the human attention system primarily consists of the alerting, orienting, and executive control networks. The alerting network is responsible for achieving and maintaining readiness for incoming information. From an information-processing perspective, it reflects the benefit of advance warning in preparing the individual for subsequent stimulus processing and response. The orienting network supports the selective allocation and shifting of attentional resources toward relevant locations or stimuli, thereby prioritizing task-relevant information. The executive control network is primarily involved in monitoring and resolving conflict among competing stimuli, responses, or task demands, particularly when irrelevant information must be inhibited to maintain goal-directed processing (Raz & Buhle, 2006). Together, these three networks represent complementary components of attentional processing: readiness for incoming information, selective allocation of attentional resources, and control of competing information during goal-directed responding.

Based on this framework, Fan et al. (2002) combined a cueing paradigm with the Flanker task to develop the Attention Network Test (ANT), which operationalizes these functions as the alerting effect, orienting effect, and executive control effect. These outcomes are reaction-time difference scores derived from specific cue or target conditions rather than indices of general response speed. The alerting effect represents the benefit of advance warning for response readiness, the orienting effect represents the benefit of directing attention to the location of an upcoming target, and the executive control effect represents the additional processing cost associated with resolving conflicting stimulus information. Accordingly, the three outcomes enable the present study to determine whether intervention-related differences are associated with attentional readiness, attentional orienting, or conflict-related executive control, rather than treating attention as a single global function.

At the intervention level, mindfulness meditation may be a candidate approach for improving attentional regulation in university students with problematic short-video use. Mindfulness meditation emphasizes consciously attending to present-moment bodily sensations, emotions, and thoughts with an open and nonjudgmental attitude (Davidson & Kaszniak, 2015). In focused-attention practice using the breath as the object of attention, individuals are required to notice mind wandering, redirect attention to the breath, and maintain attention on the current target. This cyclical process repeatedly trains the abilities to monitor attention, disengage from distracting information, and restore goal-directed attention (Dahl et al., 2015; Lindsay & Creswell, 2017).

In addition to mindfulness meditation, breathing exercises, cognitive training, and reducing digital media use have also been used to improve attentional function or regulate problematic digital media use behaviors (Ma et al., 2017; Olson et al., 2023; Shahrajabian et al., 2023). A distinctive feature of mindfulness practice is that it not only requires individuals to maintain attention on the current target but also directly involves awareness of attentional deviations and automatic reactions, together with repeated practice in redirecting attention to the current target. Therefore, the present study selected mindfulness meditation not because it was assumed to be superior to all alternative interventions, but because its core training processes have relatively direct theoretical links with attentional monitoring, distraction inhibition, conflict monitoring, and executive control.

Some intervention studies using the ANT suggest that the potential effects of mindfulness meditation may be more evident in the executive control network. Ainsworth et al. (2013) compared focused-attention meditation, open-monitoring meditation, and relaxation training and found that both meditation practices improved executive attention, whereas no similar change was observed in the relaxation control group. Kwak et al. (2020) found that, after 4 days of short-term intensive meditation training, the meditation group showed improved executive control network performance, accompanied by changes in activity in executive-control-related brain regions such as the anterior cingulate cortex and dorsolateral prefrontal cortex. Zhong et al. (2024) also found that 4 weeks of brief mindfulness training may improve the executive control network, but no clear changes were observed in the alerting and orienting networks. However, relevant meta-analyses indicate that the overall effects of mindfulness training on attention and executive control are relatively limited, and findings are not entirely consistent across different cognitive tasks, measurement indices, and components of executive function (Cásedas et al., 2020; Yakobi et al., 2021). Therefore, whether brief mindfulness meditation can produce observable changes in attention networks among university students with problematic short-video use, and whether its potential effects are concentrated in specific attention networks, require further examination.

In summary, problematic short-video use may be associated with difficulties in attentional regulation and executive control, whereas mindfulness meditation may influence related cognitive processes through sustained attentional monitoring, awareness of distraction, and attention-restoration practice. However, existing research has rarely directly examined the intervention-related plasticity of attention network function in university students with problematic short-video use, and short-term mindfulness intervention studies using the ANT to simultaneously distinguish the alerting, orienting, and executive control networks remain limited. Based on this, the present study employed a two-group pretest–posttest controlled design and the ANT to examine the potential effects of brief mindfulness meditation on the three attention networks in university students with problematic short-video use. Based on attention network theory and previous mindfulness research, the following hypotheses were proposed: H1, after controlling for baseline performance, the intervention group would show a higher alerting effect than the control group, indicating greater efficiency of the alerting network; H2, after controlling for baseline performance, the intervention group would show a higher orienting effect than the control group, indicating greater efficiency of the orienting network; H3, after controlling for baseline performance, the intervention group would show a lower executive control effect than the control group, indicating a lower cost of conflict processing.

## 2. Research Methods

### 2.1. Participants

This study used undergraduate students at Xizang University as the initial screening population. A total of 2005 screening questionnaire sets were distributed, with each set including the Smartphone Addiction Inventory (SPAI; Lin et al., 2014) and the Internet Addiction Test (IAT; Young, 1998) adapted to the context of short-video use. A total of 1930 valid questionnaire sets were ultimately collected, yielding a valid response rate of 96.26%.

In this study, the SPAI and adapted IAT were used only to identify potential participants with relatively high levels of problematic short-video use tendency, rather than to make a clinical diagnosis of short-video addiction. Scores of no less than 40 on both the SPAI and the adapted IAT were set as the operational screening criteria, and students who met these criteria were included in the pool of candidate participants.

The inclusion criteria were as follows: (1) currently enrolled undergraduate students at Xizang University; (2) actual experience of short-video use; (3) scores of no less than 40 on both the SPAI and the adapted IAT; (4) voluntary participation in the experiment and provision of informed consent; and (5) right-handedness and normal or corrected-to-normal vision. The exclusion criteria were the presence of neurological diseases, psychiatric or psychological disorders, or attention deficits that might affect performance on the attention task; current use of medications that might affect the central nervous system or cognitive performance; nicotine, caffeine, or other substance dependence; and inability to complete the pretest, intervention, or posttest in accordance with the experimental requirements. The above health conditions and relevant experiences were confirmed through participants’ self-reports.

The researchers randomly selected students from among the candidate participants who met the screening criteria and satisfied the inclusion and exclusion criteria, and invited them to participate voluntarily in the experiment. Eligible participants who agreed to take part were then randomly assigned to either the intervention group or the control group. A total of 71 undergraduate students completed all study procedures, including 38 participants in the intervention group, of whom 29 were male, and 33 participants in the control group, of whom 27 were male. All participants were Han Chinese undergraduate students. The age, sex composition, and screening scale scores of the two groups are presented in Table 1.

During the study, the intervention group received brief mindfulness meditation training, whereas the control group received no mindfulness training or other specialized attention training. This study was approved by the Ethics Committee of Xizang University. All participants provided written informed consent before the experiment began and received corresponding compensation after completing the experiment.

### 2.2. Experimental Materials and Procedure

#### 2.2.1. Attention Network Test (ANT)

**Figure 1:**
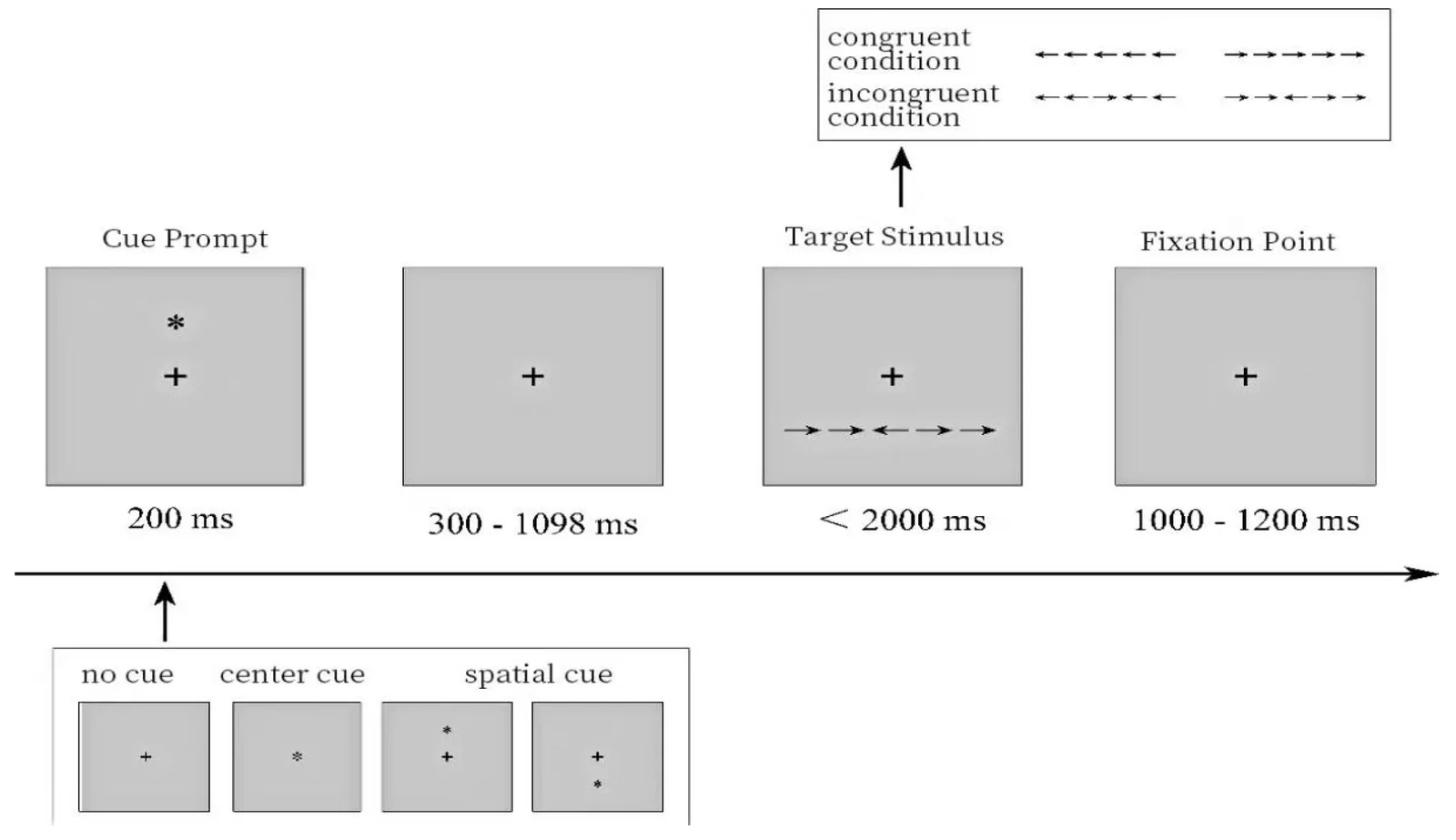
Stimuli and experiment procedure of this study. Note. “+” indicates the fixation point, and “*” indicates the cue stimulus.

The ANT was used to examine the functions of the alerting, orienting, and executive control attention networks (Figure 1). The experiment was programmed using E-Prime 2.0, and the stimuli were presented on a 17-inch laptop computer. The experiment included three cue conditions (no cue, center cue, and spatial cue) and two target stimulus conditions (congruent and incongruent). The target stimulus consisted of five horizontally arranged arrows. Each arrow subtended a visual angle of 0.58°, the visual angle between adjacent arrows was 0.06°, and the overall visual angle was 3.27°. The target stimulus was presented at a position 1.06° away from the central fixation point. In the incongruent condition, the direction of the middle arrow was opposite to that of the arrows on both sides; in the congruent condition, all five arrows pointed in the same direction. At the beginning of each trial, a cue was presented for 200 ms, followed by the fixation point “+” presented randomly for 300–1098 ms, and then the target stimulus was presented. If the participant did not respond within 2000 ms, the target stimulus disappeared, and the trial was recorded as an incorrect response. The inter-trial interval was randomly set at 1000–1200 ms. Participants were required to judge the direction of the central arrow in the target stimulus as quickly and accurately as possible (pressing the F key for left and the J key for right). The experiment included six blocks, with 108 trials in each block. Before the formal experiment, 12 practice trials were conducted. The frequency of each condition was the same within each block.

The three attention network effects were quantified using reaction-time difference scores across specific ANT conditions (Fan et al., 2002). The alerting effect was calculated as RT_no-cue − RT_center-cue. The no-cue condition provided no advance warning of the upcoming target, whereas the center-cue condition provided temporal warning without information about the target location. Therefore, the resulting difference reflects the extent to which an advance warning cue facilitates readiness for subsequent target processing; a larger alerting effect indicates a greater alerting benefit and higher efficiency of the alerting network.

The orienting effect was calculated as RT_center-cue − RT_spatial-cue. Both conditions provided advance warning, but the spatial cue additionally indicated the location at which the target would appear. The difference therefore reflects the processing benefit obtained from directing attentional resources to the relevant spatial location; a larger orienting effect indicates a greater spatial-orienting benefit and higher efficiency of the orienting network.

The executive control effect was calculated as RT_incongruent − RT_congruent. In congruent trials, the flanking arrows pointed in the same direction as the central target arrow, whereas in incongruent trials, they pointed in the opposite direction and generated response conflict. The difference therefore represents the additional processing time associated with resolving interference from competing information. In contrast to the alerting and orienting effects, a smaller executive control effect indicates a lower conflict-processing cost and higher efficiency of the executive control network. Accordingly, the three ANT outcomes index distinct aspects of attentional processing and should not be interpreted as equivalent to overall reaction time or accuracy.

#### 2.2.2. Smartphone Addiction Inventory (SPAI)

The Smartphone Addiction Inventory was developed by Lin et al. (2014) to assess individuals’ tendency toward smartphone addiction. The scale contains 26 items and includes four factors: compulsive behavior, withdrawal, tolerance, and functional impairment. The SPAI uses a 4-point scoring method, where 1 indicates “strongly disagree”, and 4 indicates “strongly agree.” The total score is obtained by adding the scores of all items, with higher scores indicating a more severe tendency toward smartphone addiction. In this study, the Cronbach’s α of this scale was 0.867.

#### 2.2.3. Adapted Internet Addiction Test (IAT)

This study used the 20-item Chinese version of the Internet Addiction Test (IAT) developed by Young (1998), and replaced “Internet” in the items with “short-video app” according to the research purpose, while keeping the remaining item structure and scoring method unchanged. This adapted scale was mainly used as a screening tool for short-video addiction tendency in this study. The scale uses a 5-point scoring method, where 1 indicates “rarely”, and 5 indicates “always.” A higher score indicates more serious problems caused by the use of short-video apps. The scale content mainly involves salience, excessive use, difficulty in control, neglect of work, neglect of social life, and anticipation. In the sample of this study, the Cronbach’s α of the adapted scale was 0.852.

#### 2.2.4. Brief Mindfulness Meditation Intervention

This study used the “JW2016 version” audio of brief mindfulness meditation training (BMM) developed by Wu et al. (2019) to train the intervention group. The intervention lasted for 4 weeks, 5 days per week, and 15 min each time. During the training period, trained research assistants played the audio in a unified way and supervised the training process, while recording participants’ attendance and training completion. Apart from the audio content, the research assistants did not provide additional meditation guidance, in order to ensure the standardization of the intervention procedure. Participants conducted focused breathing meditation according to the audio guidance and focused their attention on the breathing process. When they noticed that their attention had wandered, participants followed the audio prompts to bring their attention back to breathing. The control group received no mindfulness meditation training or other alternative intervention during the same period. This intervention used breath observation as the main form of training, emphasizing awareness of present-moment experience and active regulation of attention. All training sessions were implemented according to the same audio, fixed duration, and identical procedure to ensure consistency in the intervention process.

### 2.3. Research Design

This study employed a two-group pretest–posttest controlled design, including an intervention group and a control group. Group was treated as a between-subjects variable, and measurement time included the pretest and posttest. The study aimed to examine differences in attention network performance between university students with problematic short-video use in the intervention and control groups following brief mindfulness meditation training.

This study primarily focused on the three attention network effects measured by the Attention Network Test (ANT), namely the alerting effect, orienting effect, and executive control effect. Given that the three attention networks collectively reflect attention network function, they were simultaneously included in the overall multivariate analysis to examine whether there were overall differences between the two groups across the three attention network outcomes after controlling for baseline performance, age, and sex. Reaction time (RT) and accuracy (ACC) under the five ANT task conditions—no cue, center cue, spatial cue, congruent, and incongruent—were treated as secondary descriptive task-performance indicators to provide additional information on pre–post patterns of response speed and accuracy across specific task conditions.

### 2.4. Data Collection and Processing

Data from the attention network task were collected using E-Prime 2.0 and statistically analyzed using SPSS 23.0. First, descriptive statistics were used to present participants’ demographic characteristics, screening scale scores, and main attention network indicators. Independent-samples *t* tests were used to compare age, SPAI scores, and adapted IAT scores between the intervention and control groups, and a chi-square test was used to compare the sex composition of the two groups. These comparisons were primarily used to describe the initial characteristics of the two groups, whereas intervention-related inferences were based on statistical models that controlled for baseline performance.

To examine the overall differences between the two groups across the three attention network outcomes, multivariate analysis of covariance (MANCOVA) was conducted. Posttest alerting, orienting, and executive control effects were entered as the combined dependent variables, group was entered as the fixed factor, and the corresponding pretest scores of the three attention network effects, age, and sex were included as covariates. The overall multivariate group effect was evaluated using Pillai’s Trace.

Based on the overall multivariate analysis, exploratory follow-up analyses of covariance (ANCOVAs) were conducted separately for the alerting, orienting, and executive control effects. In each analysis, the posttest attention network effect was entered as the dependent variable, group was entered as the fixed factor, and the corresponding pretest attention network effect score, age, and sex were controlled as covariates. To control for the multiple-testing issue arising from comparisons across the three outcome indicators, the Holm method was applied to adjust the *p* values of the three follow-up ANCOVAs, and the unadjusted *p* values, Holm-adjusted *p* values, and partial η^2^ were reported simultaneously.

RT and ACC under the five ANT task conditions were treated as secondary descriptive task-performance indicators. Means and standard deviations were calculated separately for the intervention and control groups at pretest and posttest for each condition. These condition-specific data were used only to describe patterns of task performance and were not subjected to separate inferential significance tests.

## 3. Results

As shown in Table 1, no significant between-group differences were observed in age, *t*(69) = −0.379, *p* = 0.706, or sex distribution, *χ*^2^(1) = 0.321, *p* = 0.571. The intervention and control groups also did not differ significantly in SPAI scores, *t*(69) = 0.023, *p* = 0.982, or adapted IAT scores, *t*(69) = 0.073, *p* = 0.942.

To examine the overall intervention-related differences across the three attention network outcomes, a multivariate analysis of covariance (MANCOVA) was conducted. Post-intervention alerting, orienting, and executive control effects were entered simultaneously as dependent variables, with group as the independent variable. The corresponding baseline attention network scores, age, and sex were included as covariates. The overall multivariate effect of group was not statistically significant, Pillai’s Trace = 0.114, *F*(3, 62) = 2.654, *p* = 0.056, partial η^2^ = 0.114.

Given the nonsignificant overall multivariate effect, follow-up ANCOVAs were treated as exploratory. Each analysis controlled for the corresponding baseline score, age, and sex, and Holm adjustment was applied across the three outcome-specific comparisons. As shown in Table 2, no significant between-group difference was found for the alerting effect, *F*(1, 66) = 0.365, *p* = 0.548, Holm-adjusted *p* = 1.000, partial η^2^ = 0.006, or for the orienting effect, *F*(1, 66) = 0.413, *p* = 0.523, Holm-adjusted *p* = 1.000, partial η^2^ = 0.006.

In contrast, the executive control effect showed a significant between-group difference, *F*(1, 66) = 7.998, *p* = 0.006, Holm-adjusted *p* = 0.019, partial η^2^ = 0.108. After adjustment for baseline executive control performance, age, and sex, the estimated post-intervention executive control effect was lower in the intervention group than in the control group (52.490 vs. 70.870). Because lower executive control effect scores indicate lower conflict-processing costs, this exploratory finding suggests an outcome-specific difference in executive control performance. However, because the overall multivariate effect was not statistically significant, the finding should be interpreted cautiously and should not be regarded as evidence of a general improvement across the three attention networks.

Condition-specific RT and ACC were examined descriptively as secondary task-performance indicators. Descriptive statistics for the five ANT conditions are presented in Supplementary Table S1. Across conditions, the pattern of pre–post changes did not indicate a consistent improvement in response speed or accuracy in the intervention group relative to the control group.

## 4. Discussion

This study examined the potential effects of brief mindfulness meditation on attention network function in university students with problematic short-video use. After controlling for baseline attentional performance, age, and sex, the overall multivariate group effect across the three attention networks did not reach statistical significance. Exploratory follow-up analyses showed that only the executive control effect differed significantly between the two groups, and this difference remained statistically significant after Holm correction, whereas the alerting and orienting effects did not differ significantly between the groups. Therefore, H1 and H2 were not supported, whereas H3 received only exploratory support. Overall, the current findings do not support the conclusion that brief mindfulness meditation can comprehensively improve attention network function, but the exploratory results suggest that the intervention group may exhibit a lower cost of conflict processing.

The executive control effect is calculated as the RT difference between incongruent and congruent conditions and reflects the additional cost generated by conflict processing; a lower effect value generally indicates a lower conflict-processing cost (Fan et al., 2002; Raz & Buhle, 2006). In the present study, the intervention group showed a lower executive control effect than the control group after controlling for covariates, which is, to some extent, consistent with previous findings suggesting that mindfulness training may improve executive attention or executive control (Ainsworth et al., 2013; Kwak et al., 2020). In contrast, no significant between-group differences were observed for the alerting or orienting effects, which is relatively consistent with the findings of Zhong et al. (2024), suggesting that the potential effects of short-term mindfulness training may be more concentrated on conflict monitoring and interference inhibition rather than extending to general alertness preparation or spatial orienting processes.

This finding may be related to the attentional regulation processes involved in mindfulness practice. During focused-attention practice, individuals are required to maintain the current goal, detect attentional deviations, and redirect attention, which may train attentional monitoring and the ability to disengage from distracting information (Tang et al., 2015). Relevant randomized controlled studies have also found that short-term mindfulness training may influence the functional connectivity of the dorsolateral prefrontal cortex and frontoparietal control network, providing neurofunctional evidence for its potential effects on executive control processes (Taren et al., 2017). However, the present study did not directly measure these psychological or neural processes; therefore, the above mechanistic explanation remains theoretical speculation.

The findings of this study can also be understood from the perspective of the I-PACE model of specific Internet-use disorders. This model proposes that the development and maintenance of problematic Internet use involve interactions among personal characteristics, affective and cognitive responses, cue reactivity, craving, and executive control, with inhibitory control playing an important role in regulating automatic responses and craving (Brand et al., 2019). University students with problematic short-video use may be frequently exposed to rapidly switching, immediately rewarding, and continuously updated content streams (Zhang et al., 2024), whereas mindfulness practice repeatedly trains awareness of mind wandering and the ability to redirect attention to the current target (Hasenkamp et al., 2012). From this perspective, the exploratory difference in executive control observed in the present study may reflect a potential effect of mindfulness training on goal-directed attention and conflict-processing-related processes rather than a comprehensive improvement in general attentional function. However, the present study did not measure cue reactivity, craving, or actual short-video use behavior, and the overall multivariate effect did not reach statistical significance; therefore, this interpretation requires further verification.

Descriptive patterns of RT and ACC across the five ANT conditions did not indicate a consistent improvement in condition-specific response speed or accuracy in the intervention group relative to the control group. The executive control effect is a relative index calculated from the RT difference between incongruent and congruent conditions; therefore, a between-group difference in this effect does not imply that the intervention group responded faster or more accurately across all task conditions (Galvao-Carmona et al., 2014). Previous studies have shown that the effects of mindfulness interventions vary across different cognitive indicators, and their effects on reaction time or latency-based measures of executive function are particularly inconsistent (Yakobi et al., 2021; Zainal & Newman, 2024). Therefore, the current exploratory finding is more appropriately interpreted as a specific difference in conflict-processing cost rather than as a general improvement in response speed or accuracy across individual task conditions.

Different types of problematic Internet use may involve some shared mechanisms, such as reinforcement-driven repetitive use, reward processing, and weakened inhibitory control (Moretta et al., 2022). Research on Internet gaming disorder has shown that mindfulness interventions may exert their effects by reducing gaming craving, modifying maladaptive gaming cognitions, and influencing neural activity related to cognitive control and reward processing (Li et al., 2018; Ni et al., 2024; Xu et al., 2025). These findings suggest that enhancing awareness of automatic responses and top-down regulatory capacity may represent a common mechanism through which mindfulness interventions affect different types of problematic digital media use.

However, short-video platforms are characterized by algorithmic recommendation, continuous scrolling, and rapid content switching, enabling users to remain continuously exposed to updated content with relatively low operational effort (Mou et al., 2021; Wang et al., 2026). Therefore, the potential effects of mindfulness interventions on problematic short-video use may involve automatic attentional shifting, interference monitoring, and conflict processing to a greater extent, whereas in Internet gaming disorder, they may additionally involve gaming craving, maladaptive gaming cognitions, and reward processing. Because the present study did not include other problematic Internet-use groups as comparison groups, whether the current findings are specific to the short-video context remains to be further examined through direct comparative research.

### 4.1. Study Limitations

This study has several limitations. First, the SPAI and adapted IAT were primarily used to identify participants with relatively high levels of problematic short-video use tendency rather than for clinical diagnosis. Although the adapted IAT demonstrated good internal consistency in the present sample, its measurement applicability in the context of problematic short-video use requires further validation; therefore, the interpretation of participant screening and related findings should remain cautious. Participants’ neurological diseases, psychological or psychiatric disorders, attention deficits, relevant medication use, and substance dependence were mainly confirmed through self-report and were not further verified using standardized clinical instruments. In addition, this study did not collect detailed information on participants’ majors, academic years, years of short-video use, daily use duration, or weekly use frequency. Therefore, the sample characteristics could not be further described according to participants’ majors and academic years, and potential differences associated with the actual intensity and duration of short-video use could not be controlled. These factors may affect the interpretation of the sample characteristics and study findings.

Second, the duration and intensity of the intervention in this study were relatively limited and may have been insufficient to produce stable changes in the alerting network, orienting network, or general task performance. This study employed a no-intervention control condition and did not include an active control group matched in terms of training duration, mode of instruction, and degree of researcher contact; therefore, it is difficult to distinguish the specific effects of mindfulness practice from nonspecific effects such as relaxation, expectancy, or study participation. Both groups completed the ANT repeatedly, and posttest performance may also have been influenced by practice effects or fatigue (Bartels et al., 2010; Goldberg et al., 2015). In addition, this study did not directly measure mindfulness state, attentional engagement, practice quality, or adherence, which limits the assessment of intervention implementation quality and mechanisms of action.

Finally, the sample size of this study was relatively limited, and all participants were recruited from a single university, with relatively concentrated sex distribution and cultural background, which may limit statistical power and the generalizability of the findings. The overall multivariate effect across the three attention networks did not reach statistical significance, and the difference in executive control emerged only from exploratory follow-up analysis; although it remained significant after Holm correction, it should still be regarded as a preliminary finding requiring replication. In addition, this study relied solely on behavioral indicators from the ANT and did not incorporate neurophysiological measures such as EEG, ERP, or fMRI; therefore, the relevant cognitive and neural mechanisms cannot be directly determined.

### 4.2. Future Research Directions

Future research should expand the sample size and sources, adopt preregistered randomized controlled trial designs, and include an active control group matched with the mindfulness group in terms of training duration, mode of instruction, and degree of researcher contact, in order to replicate the current findings and distinguish the specific effects of mindfulness training from nonspecific effects. At the same time, different training durations, practice frequencies, and levels of adherence could be compared to examine whether intervention effects vary according to training dosage.

Future studies should also use specifically validated measures of problematic short-video use and combine them with duration of use, frequency of use, years of continuous use, and objective screen-use records to improve measurement accuracy. Research could also compare groups with problematic short-video use, Internet gaming disorder, and problematic smartphone use, and incorporate EEG, ERP, or fMRI, as well as measures of mindfulness state, cue-induced craving, self-control, and actual use behavior, to examine the shared and specific mechanisms of mindfulness interventions. In particular, it is necessary to examine whether changes in attention networks can predict reductions in problematic use symptoms or objective use time, thereby determining whether changes in behavioral task indicators have practical intervention significance.

## 5. Conclusions

Overall, this study did not find a significant overall effect of brief mindfulness meditation on the three attention network functions in university students with problematic short-video use. After controlling for baseline attentional performance, age, and sex, neither the alerting effect nor the orienting effect showed significant between-group differences. Exploratory follow-up analyses showed that the intervention group had a lower executive control effect than the control group, and this difference remained statistically significant after Holm correction, suggesting that brief mindfulness meditation may be associated with a lower cost of conflict processing. Descriptive patterns of RT and ACC across the five ANT conditions did not indicate a consistent improvement in task performance.

Therefore, the current findings do not support the conclusion that brief mindfulness meditation can comprehensively improve attention network function or general task performance in university students with problematic short-video use, but provide only preliminary exploratory evidence that it may influence conflict processing related to executive control. Given that the overall multivariate effect did not reach statistical significance, this finding should still be interpreted cautiously and requires further verification in studies with larger samples and more rigorous control conditions.

## Figures and Tables

**Table 1:** Demographic Characteristics and Screening Measures of the Intervention and Control Groups.

Variable	Intervention Group	Control Group	Statistic	*p*
Age	20.368 ± 0.883	20.455 ± 1.034	*t*(69) = −0.379	0.706
Sex, male/female	29/9	27/6	*χ*^2^(1) = 0.321	0.571
SPAI score	68.605 ± 11.744	68.545 ± 10.429	*t*(69) = 0.023	0.982
Adapted IAT score	54.737 ± 10.820	54.545 ± 11.130	*t*(69) = 0.073	0.942

Note. Age, SPAI, and adapted IAT scores are presented as *M* ± *SD*. Sex is reported as male/female counts. SPAI = Smartphone Addiction Inventory; IAT = Internet Addiction Test.

**Table 2:** Descriptive Statistics and Exploratory Baseline-Adjusted ANCOVA Results for Attention Network Outcomes.

Attention Network	Intervention Group Pretest	Intervention Group Posttest	Control Group Pretest	Control Group Posttest	*F*(1, 66)	*p*	Holm-Adjusted *p*	Partial η^2^
Alerting	7.633 ± 13.059	8.538 ± 23.526	6.474 ± 16.849	5.983 ± 14.379	0.365	0.548	1.000	0.006
Orienting	41.321 ± 21.021	43.033 ± 29.935	40.754 ± 22.729	38.418 ± 21.732	0.413	0.523	1.000	0.006
Executive control	69.625 ± 24.020	52.456 ± 32.216	70.769 ± 19.290	70.911 ± 18.411	7.998	0.006	0.019	0.108

Note. Values are presented as *M* ± *SD*. Follow-up ANCOVAs were exploratory and controlled for the corresponding baseline score, age, and sex. Holm-adjusted *p* values account for the three outcome-specific comparisons. Lower executive control effect scores indicate lower conflict-processing costs.

## Data Availability

The data that support the findings of this study are not publicly available due to participant privacy/ethics restrictions, but are available from the corresponding author upon reasonable request.

## Supplementary Materials

The following supporting information can be downloaded at: https://www.mdpi.com/article/10.3390/bs16091487/s1, Table S1: Descriptive Statistics for RT and ACC Across the Five ANT Conditions.
