# Supplementary material for: The Effect of Brief Mindfulness Meditation on Attention Networks in University Students with Problematic Short-Video Use
Source: Behav Sci (Basel). 2026 Aug 25;16(9):1487. doi: 10.3390/bs16091487 (PMC13603608; doi:10.3390/bs16091487)
Supplement: Supplementary file 1 [file behavsci-16-01487-s001.zip › behavsci-4381834-supplementary.pdf]

Supplementary Table S1: Descriptive Statistics for RT and ACC Across the Five ANT Conditions

ANT condition	Intervention group pretest	Intervention group posttest	Control group pretest	Control group posttest
RT（ms）				
no cue	579.569±72.682	574.934±108.410	583.328±75.912	565.485±94.823
center cue	571.937±72.628	566.396±115.814	576.854±77.801	559.502±95.875
spatial cue	530.616±75.715	523.363±108.007	536.010±80.705	521.084±96.342
congruent condition	529.491±66.708	523.802±104.285	529.336±75.030	520.205±89.789
Incongruent condition	599.116±80.528	576.258±111.698	600.105±81.230	591.116±85.562
ACC				
no cue	0.967±0.042	0.976±0.017	0.973±0.031	0.969±0.023
center cue	0.970±0.033	0.971±0.022	0.975±0.023	0.963±0.025
spatial cue	0.976±0.036	0.980±0.013	0.983±0.020	0.972±0.028
congruent condition	0.980±0.036	0.983±0.014	0.983±0.027	0.974±0.026
Incongruent condition	0.963±0.037	0.973±0.025	0.963±0.032	0.959±0.033

Note. Values are presented as $M \pm SD$. RT = reaction time; ACC = accuracy; ANT = Attention Network Test. Condition-specific RT and ACC were treated as secondary descriptive task-performance indicators and were not subjected to separate inferential significance tests.
